# Multimodal NIR–Micro-XRF Phenotyping Captures Complementary Chemical and Elemental Variation Across All South American *Nothofagus*

**DOI:** 10.3390/plants15213374

**Published:** 2026-11-01

**Authors:** Freddy Mora-Poblete, Cristian Echeverría, Milenka Rivera, Jordán Pérez-Martínez, Ricardo Cabeza

**Affiliations:** 1 Institute of Biological Sciences, University of Talca, Av. Lircay, Talca 3461334, Chile; milenka.rivera@utalca.cl (M.R.); joperez21@alumnos.utalca.cl (J.P.-M.); 2 Laboratory of Landscape Ecology, Faculty of Forest Sciences, University of Concepción, Concepción 4030000, Chile; crisecheverria@udec.cl; 3 Plant Nutrition Laboratory, Department of Crop Sciences, Faculty of Agricultural Sciences, University of Talca, Av. Lircay, Talca 3460000, Chile; rcabeza@utalca.cl

**Keywords:** ex situ conservation, conservation phenomics, common garden, plant biodiversity, near-infrared spectroscopy, X-Ray fluorescence

## Abstract

Rapid characterization of conserved tree germplasm can strengthen ex situ conservation as global change intensifies pressures on forest biodiversity. We evaluated whether NIR–micro-XRF phenotyping can resolve complementary signatures across all ten South American *Nothofagus* species (*N. alessandrii*, *N. alpina*, *N. antarctica*, *N. betuloides*, *N. dombeyi*, *N. glauca*, *N. macrocarpa*, *N. nitida*, *N. obliqua*, and *N. pumilio*) and the natural hybrid *N. × leoni* maintained in a common garden. We combined phenetic analysis, element-resolved inference, repeated cross-validated PLS-DA, multimodal fusion, and unsupervised O2PLS. Predictive analyses focused on nine species represented by 12 independent trees each (*n* = 108); *N. nitida*, represented by one tree with three technical measurements, was retained for descriptive analyses but excluded from predictive modelling. Full-spectrum XRF outperformed NIR (accuracy 0.822 ± 0.056 versus 0.701 ± 0.064), while block-equalized fusion yielded the highest accuracy (0.842 ± 0.040) but only modest gains over XRF alone. O2PLS showed that 45.8% of NIR and 52.1% of XRF variation belonged to shared structure, which concentrated stronger taxon-associated organization (PERMANOVA R^2^ = 0.605) than sensor-specific components. Fe-, Zn-, Ca-, and Mn-associated signals showed the strongest taxon differentiation. With validation in independent collections and natural populations, this multimodal framework could support taxonomic verification, phenotypic characterization, and conservation auditing of living collections.

## 1. Introduction

Living collections are an important component of ex situ plant conservation, but their conservation value depends on reliable taxonomic identity and effective characterization of the biological diversity they contain. This diversity spans complementary taxonomic, functional and evolutionary dimensions, with intraspecific phenotypic variation adding an important layer that is often underrepresented in comparative assessments [1,2]. These challenges are particularly relevant for long-lived trees, for which incomplete provenance information and restricted representation can constrain collection assessment and management. High-dimensional spectral phenotypes can help address these challenges by providing complementary information on taxonomic identity and phenotypic variation, provided that their biological interpretation is kept distinct from their predictive performance [3,4,5]. Against this background, scalable phenotypic characterization of taxonomically comprehensive ex situ collections can provide a baseline for conserving tree lineages exposed to rapidly changing environments.

Leaf reflectance spectra constitute integrative phenotypes rather than measurements of a single predefined trait. In the near-infrared and short-wave infrared regions, variation arises from broad overtone and combination absorptions involving O-H, C-H and N-H bonds, together with physical scattering associated with tissue and particle structure. Consequently, NIR spectra integrate covariation in water-associated properties, carbohydrates, lignin, cellulose, proteins, lipids and nitrogen-related chemistry [6,7]. Leaf spectra can distinguish closely related taxa, retain phylogenetic signal across seed plants and recover genetic identity under contrasting environmental conditions [8,9,10]. Proof-of-concept studies have further shown that spectroscopy can predict molecular-marker-defined genetic groups below the species level in both crop and forest-tree material [11,12]. Nevertheless, spectral phenotypes are not environmentally invariant: they integrate genotype, environment and their interaction [9].

X-Ray fluorescence provides a physically and biologically distinct spectral phenotype. Rather than measuring molecular absorption and scattering, XRF detects element-characteristic secondary X-Rays emitted after inner-shell excitation. The plant ionome is an integrated phenotype shaped by soil and rhizosphere conditions, selective uptake, transport, storage and homeostatic regulation under genetic and developmental control [13,14]. Benchtop and portable XRF systems can rapidly profile multiple elements in plant material, but signal magnitude depends on sample presentation, particle size, thickness, geometry and calibration; standardized drying, milling and acquisition are therefore essential [15,16,17]. XRF ionomics has revealed coordinated elemental patterns in plant and herbarium material, yet the capacity of whole XRF spectra to encode forest-tree taxon identity and complement NIR-derived molecular-compositional and scattering-related phenotypes remains comparatively underexplored [18].

Previous plant studies have combined NIR and XRF primarily for quantitative foliar analysis rather than taxon discrimination. Comino et al. combined FT-NIR and portable energy-dispersive XRF measurements from dried, ground olive leaves and evaluated low- and mid-level data fusion for nutrient prediction and crop nutritional diagnosis [19]. This work established the analytical complementarity of the two modalities, but it did not address whether paired NIR and micro-XRF measurements obtained from the same standardized material recover shared versus modality-specific taxon-associated structure. To our knowledge, this question remains underexplored in long-term ex situ tree collections.

A long-established common garden provides a stringent setting in which to evaluate this complementarity. Sampling natural populations would confound persistent taxon differentiation with immediate plastic responses to local environments. Prolonged growth at one site attenuates, but does not eliminate, contemporary environmental heterogeneity and allows persistent differences to be interpreted as phenotypic differentiation maintained under common conditions rather than as purely genetic effects. Common-garden spectroscopy in *Populus fremontii* has shown that genetic identity, environment and their interaction remain jointly detectable in leaf reflectance [9]. The relevant question is therefore which dimensions of biological variation remain detectable after prolonged exposure to a shared environment and whether NIR and XRF recover nonredundant components of that variation.

South American *Nothofagus* is a conservation-relevant test system because its taxa encompass deciduous and evergreen strategies, occur across contrasting temperate environments and include a reticulate evolutionary history in which hybridization, introgression and plastid capture can blur species boundaries [20,21,22]. The study set includes the threatened Chilean endemics *Nothofagus alessandrii*, *N. glauca* and *N. macrocarpa* [23]. *N. alessandrii* is particularly range-restricted and persists in fragmented forest remnants exposed to recurrent fire and invasion by *Pinus radiata* [24,25]. Long-established living collections are therefore valuable for conservation research, although incomplete provenance documentation and restricted within-species representation can limit how well they capture diversity in the wild [26].

Here, we analyzed NIR and laboratory micro-XRF spectra acquired from the same homogenized leaf samples collected from the ten South American *Nothofagus species* represented in the collection plus the natural hybrid *N. × leoni*. We addressed three linked objectives: (i) determine whether NIR and XRF recover reproducible but non-equivalent taxon-level spectral structure; (ii) quantify the element-associated basis and predictive performance of XRF discrimination relative to NIR; and (iii) test whether low-level multimodal fusion provides consistent gains over the strongest single modality. Exploratory analyses additionally evaluated broad organization by leaf habit and subgeneric affiliation, while explicitly recognizing that these attributes are confounded with taxon identity in the present collection. An additional unsupervised two-way orthogonal partial least squares (O2PLS) analysis decomposed shared and modality-specific NIR-XRF variation and evaluated where taxon-associated structure was concentrated. Together, these analyses test the ability of paired spectral modalities to characterize taxon-associated variation within an ex situ living collection, rather than to establish general taxonomic verification across populations.

Throughout this study, we use the term spectral phenotype operationally to denote the high-dimensional multivariate profile generated from standardized plant material, which integrates molecular-compositional, scattering-related, or element-associated information. It is not used as a synonym for a directly measured conventional morphological or physiological trait. Taxon discrimination refers to classification within the sampled collection, whereas taxonomic verification implies an application that would require independent validation.

## 2. Results

### 2.1. NIR and XRF Reveal Distinct, Modality-Specific Taxonomic Structure

Although the aligned analytical matrix contained 117 sample units, these did not represent 117 independent biological observations. Predictive analyses used 108 independent trees in the primary balanced dataset (nine non-hybrid species, 12 trees each) and 114 independent trees in the exploratory dataset including *N. × leoni*. The three *N. nitida* spectra originated from one biological tree and were retained only for descriptive taxon-level analyses.

**Figure 1:**
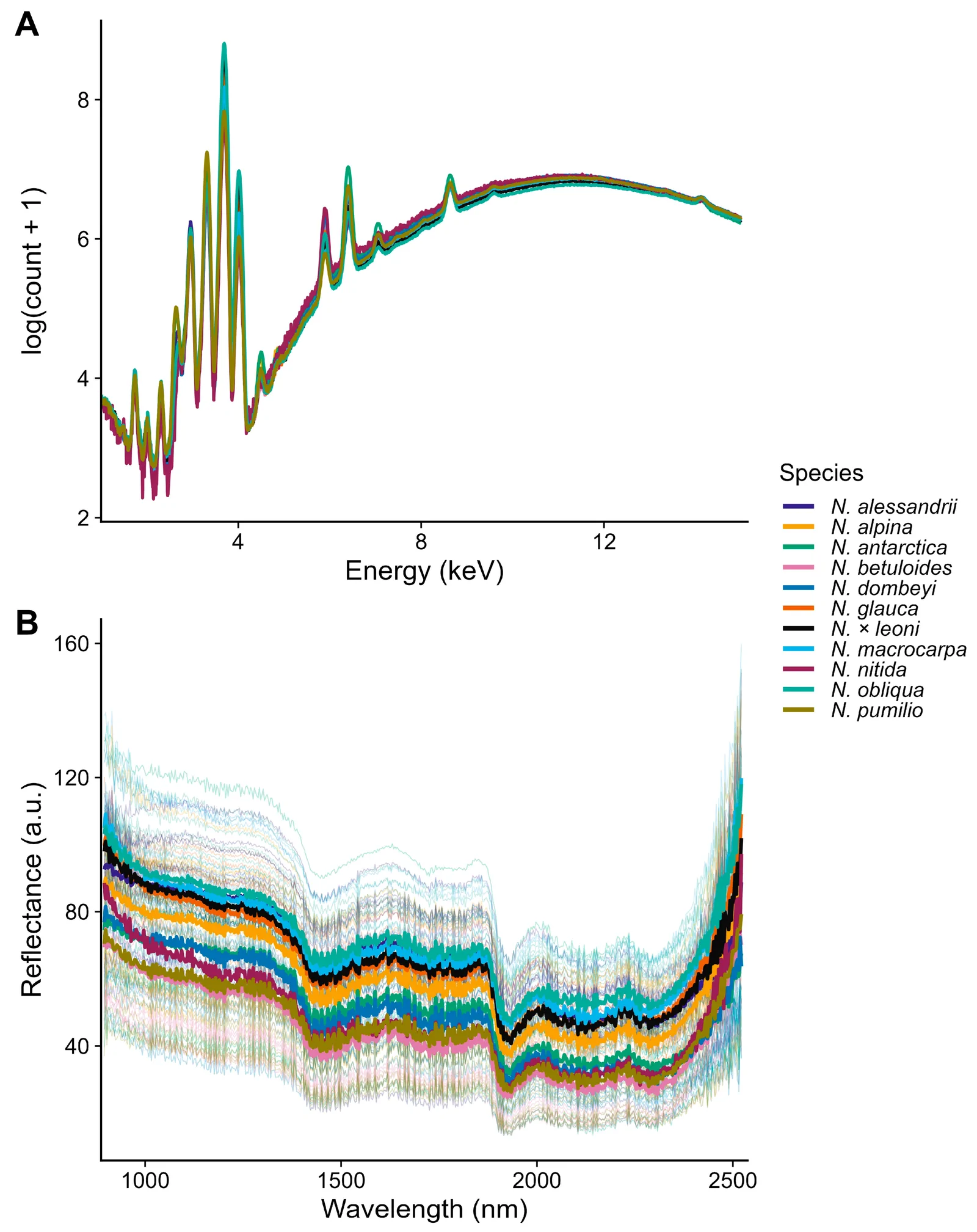
Contrasting XRF and NIR spectral phenotypes across the 11 sampled taxa. (**A**) Species-mean micro-XRF profiles, expressed as log(count + 1), from 1 to 15 keV. Each coloured line represents a taxon mean. (**B**) Raw NIR reflectance profiles across approximately 896–2515 nm, coloured by taxon. Each line represents one retained analytical unit before conversion to pseudoabsorbance and SG1 + SNV preprocessing.

XRF and NIR produced physically distinct spectral phenotypes (Figure 1). XRF spectra were characterized by narrow, element-associated peaks, most prominent below 9 keV, superimposed on a broad continuum. By contrast, raw NIR spectra displayed broad, strongly overlapping absorption features together with marked among-individual variation in baseline reflectance and spectral amplitude. These profiles are consistent with XRF capturing discrete elemental emission features and NIR capturing distributed molecular absorption and scattering responses.

**Figure 2:**
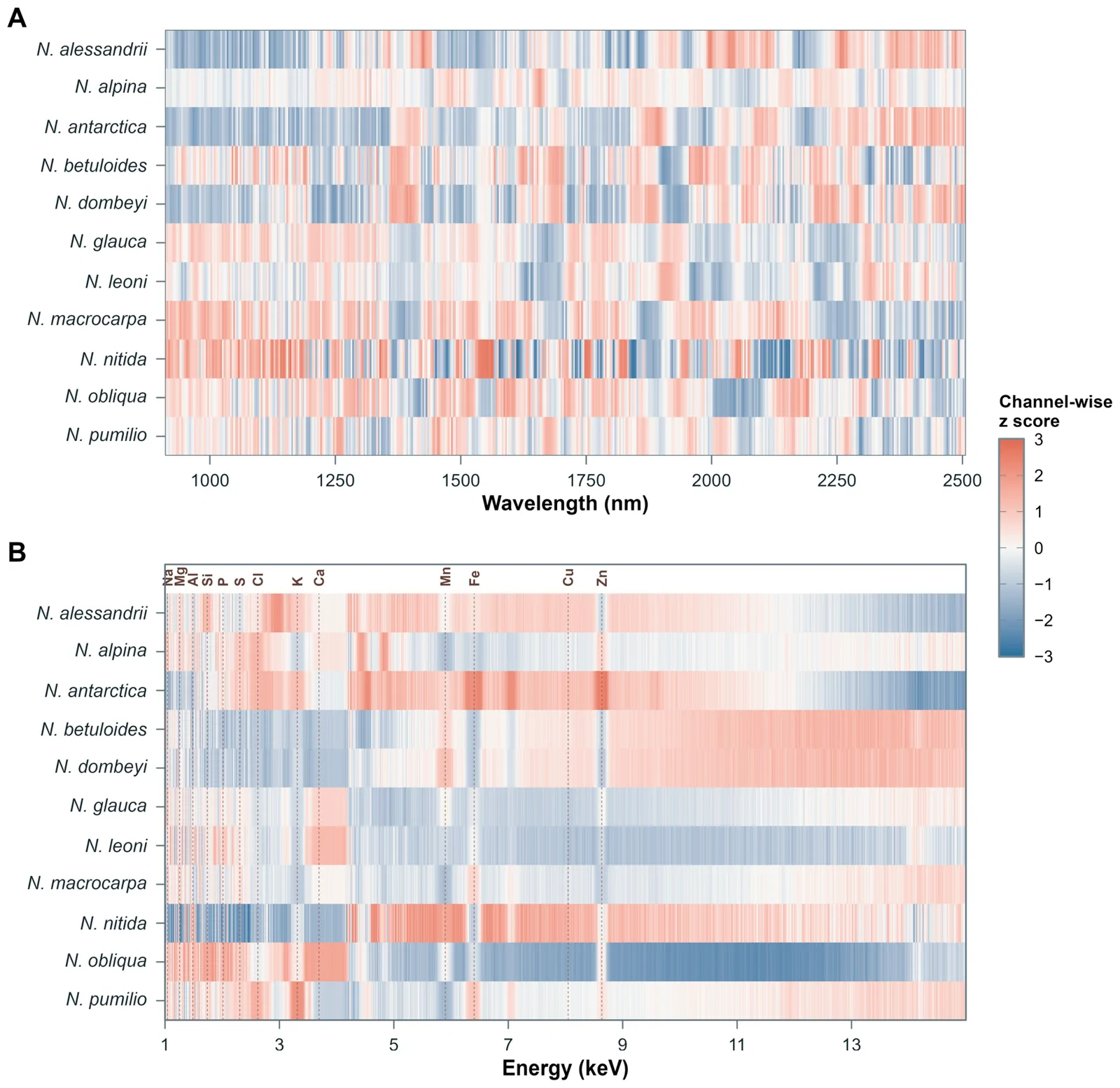
Species-mean spectral heatmaps for the 11 sampled taxa. (**A**) Channel-wise standardized taxon means from the SG1 + SNV-preprocessed NIR predictor matrix. (**B**) Channel-wise standardized taxon means from log(count + 1)-transformed XRF spectra spanning 1–15 keV; dotted lines indicate theoretical peak positions of candidate elements. The common colour scale denotes z-scores from −3 to +3, with standardization performed separately for each channel within each modality.

**Figure 3:**
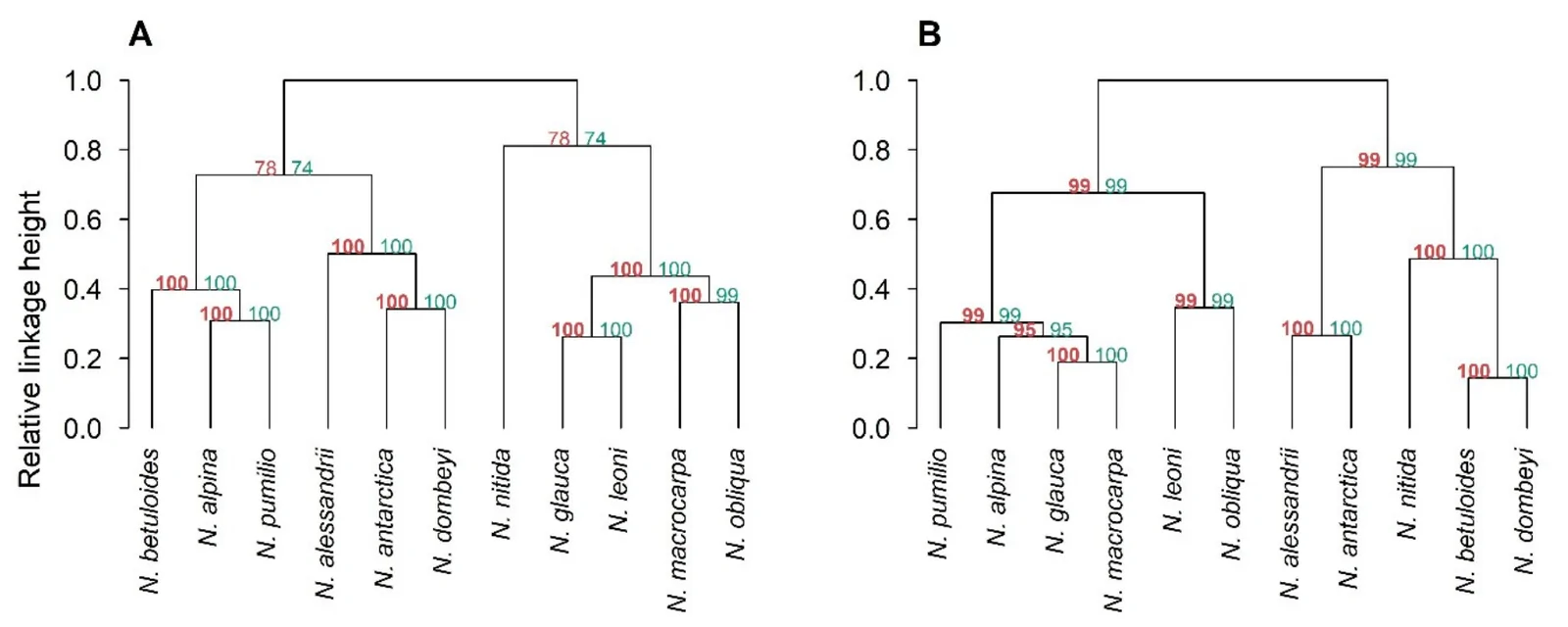
Modality-specific phenetic structure of the 11 sampled taxa. Dendrograms were obtained from Euclidean distances among channel-wise standardized taxon means using Ward.D2 clustering for (**A**) NIR and (**B**) XRF. Linkage heights were rescaled to 0–1 within each modality and should therefore be interpreted comparatively within, rather than between, panels. Red values indicate approximately unbiased (AU) support and green values indicate conventional bootstrap probability (BP) support, expressed as percentages and estimated from 10,000 bootstrap replications using the multiscale procedure implemented in pvclust. AU values ≥ 95% are shown in bold.

Species-mean heatmaps showed structured interspecific variation in both modalities, but the taxonomic arrangements differed (Figure 2 and Figure 3). In the NIR dendrogram, *N. × leoni* clustered with *N. glauca* (AU = 100, BP = 100), *N. alpina* with *N. pumilio* (AU = 100, BP = 100), *N. antarctica* with *N. dombeyi* (AU = 100, BP = 100), and *N. macrocarpa* with *N. obliqua* (AU = 100, BP = 99). In the XRF dendrogram, *N. × leoni* instead clustered with *N. obliqua* (AU = 99, BP = 99), while the pairs *N. glauca*-*N. macrocarpa*, *N. alessandrii*-*N. antarctica* and *N. betuloides*-*N. dombeyi* each received AU = 100 and BP = 100. Several deeper NIR nodes had lower support (AU/BP = 78/74), whereas the two principal deeper XRF partitions were strongly supported (AU/BP = 99/99). Thus, both modalities resolved well-supported terminal affinities, but those affinities and the higher-order phenetic structure were modality specific. Because linkage heights were independently rescaled to 0–1 within each modality, dendrogram branch lengths and heights should not be compared directly between NIR and XRF.

At the taxon-mean level, leaf habit accounted for 18.8% of NIR, 33.8% of XRF and 29.8% of feature-scaled fusion dissimilarity. Exact randomization supported leaf-habit-associated organization for XRF (R^2^ = 0.338, *p* = 0.0121) and fusion (R^2^ = 0.298, *p* = 0.0121), whereas the corresponding NIR pattern was weaker and did not reach the 0.05 threshold (R^2^ = 0.188, *p* = 0.0545). PERMDISP showed no evidence of unequal multivariate dispersion for NIR (*p* = 0.412), XRF (*p* = 0.406) or fusion (*p* = 0.848). At the species-mean level, subgeneric affiliation (Lophozonia versus *Nothofagus*, excluding the singleton Fuscospora taxon *N. alessandrii*) accounted for 28.4%, 38.9% and 36.1% of NIR, XRF and feature-scaled fusion dissimilarity, respectively (*p* = 0.0156, 0.0072 and 0.0080). PERMDISP did not detect dispersion differences for NIR (*p* = 0.0981), XRF (*p* = 0.4209) or fusion (*p* = 0.2272), although the comparatively low NIR *p*-value warrants caution in interpreting the NIR subgenus contrast. Because both leaf habit and subgenus were invariant within taxa, these results describe broad group-level spectral organization rather than effects independent of taxonomic identity. These PERMANOVA analyses used one mean spectral profile per taxon and therefore quantify broad among-taxon organization only; they do not estimate individual-tree effects or within-taxon variation.

### 2.2. Element-Resolved XRF Variation Provides an Interpretable Basis for Taxon Discrimination

**Figure 4:**
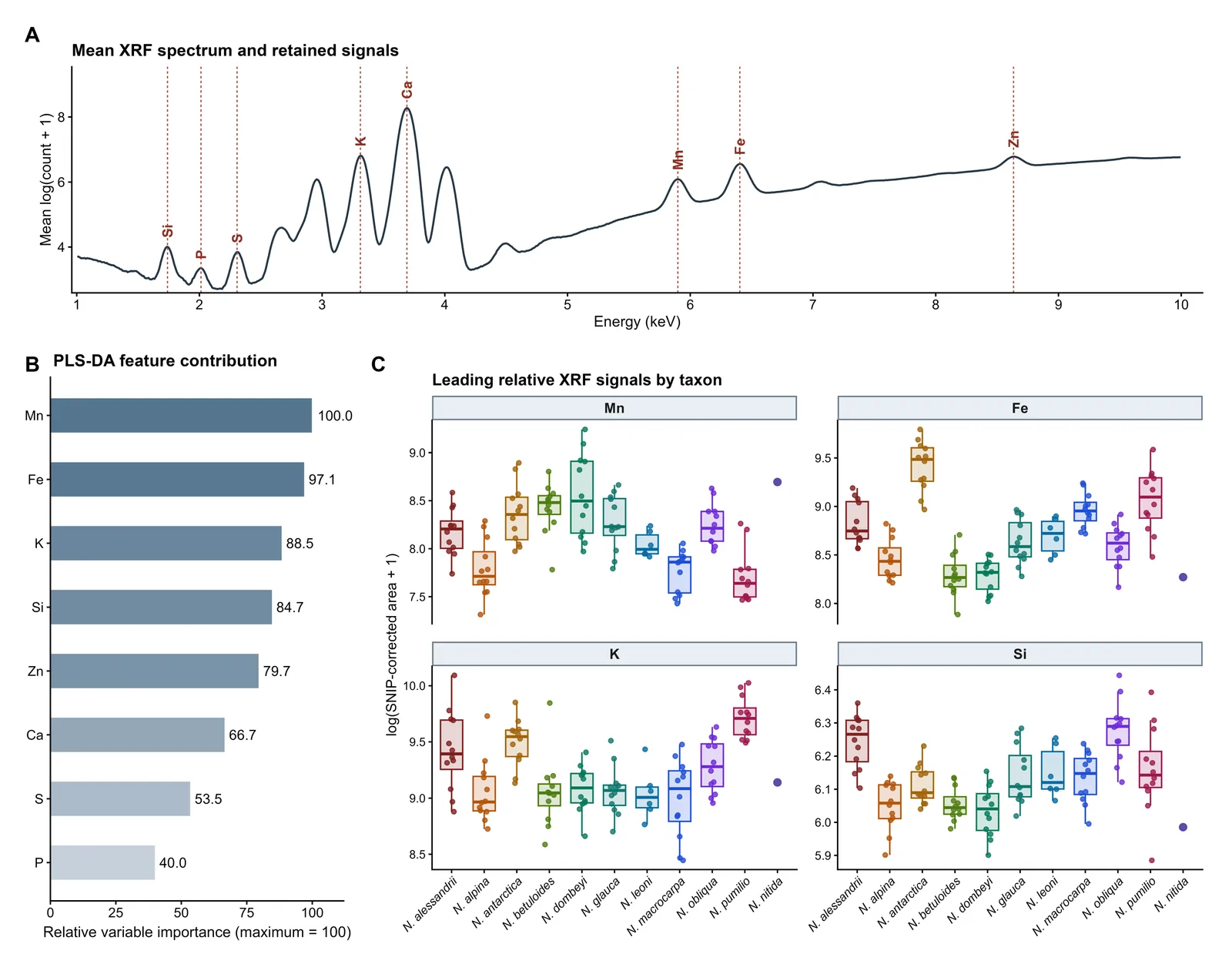
Spectral and element-resolved basis of XRF taxon discrimination. (**A**) Mean log(count + 1)-transformed XRF spectrum over the displayed 1–10 keV range, with theoretical peak positions of the eight retained element-associated signals. (**B**) Relative variable importance in the eight-signal PLS-DA model, scaled to a maximum of 100. (**C**) Distributions by taxon of the four highest-ranked statistics-sensitive nonlinear iterative peak-clipping (SNIP) background-corrected, log(area + 1)-transformed signals. For *N. nitida*, the single point represents the mean of three analytical replicates. Values are relative XRF signals rather than calibrated tissue concentrations.

Following analytical quality control and SNIP background correction, eight element-associated signals were retained for comparative analysis: Si, P, S, K, Ca, Mn, Fe and Zn (Figure 4A). Candidate peaks for Na, Mg, Al and Cu were not sufficiently resolved and were excluded. Cl was also excluded because its characteristic K-shell X-Ray emission overlaps with rhodium L-shell emission from the X-Ray tube, precluding reliable attribution.

Welch ANOVA indicated taxon effects for all eight signals after Benjamini–Hochberg adjustment (Table 1). Effect sizes were largest for Fe (η^2^ = 0.720), Zn (η^2^ = 0.691), Ca (η^2^ = 0.575) and Mn (η^2^ = 0.533), followed by Si (η^2^ = 0.487), K (η^2^ = 0.466), S (η^2^ = 0.384) and P (η^2^ = 0.198). Evidence was weakest for P (BH-adjusted *p* = 0.0439), and its taxon effect was not significant in the balanced nine-species Welch sensitivity analysis (BH-adjusted *p* = 0.0581). By contrast, taxon effects for Si, S, K, Ca, Mn, Fe and Zn were consistently supported by Welch ANOVA, Kruskal–Wallis tests and the balanced sensitivity analysis.

The PLS-DA model based on the eight retained signals selected six latent components and achieved a repeated-cross-validation accuracy of 0.598 ± 0.058 (κ = 0.551 ± 0.065). In the balanced nine-species sensitivity analysis, accuracy was 0.628 ± 0.057 (κ = 0.581 ± 0.064). Mn, Fe, K and Si were the four most influential signals (relative importance = 100.0, 97.1, 88.5 and 84.7, respectively; Figure 4B), and their taxon-level distributions are shown in Figure 4C. Across the 60 assessment partitions, mean taxon sensitivity was highest for *N. antarctica* (0.988), followed by *N. obliqua* (0.846) and *N. alpina* (0.817), but was very low for *N. betuloides* (0.104), *N. glauca* (0.067) and *N. × leoni* (0.000). The reduced signal set therefore retained substantial but incomplete discriminatory information: within the same balanced nine-species framework, its accuracy was 0.628 compared with 0.822 for the full XRF spectrum. The lower performance of the eight-signal model relative to full-spectrum XRF indicates that the retained integrated peak areas do not capture all taxon-discriminating information present in the complete spectrum.

### 2.3. Full-Spectrum XRF Outperforms NIR, with Incremental Gains from Multimodal Fusion

**Figure 5:**
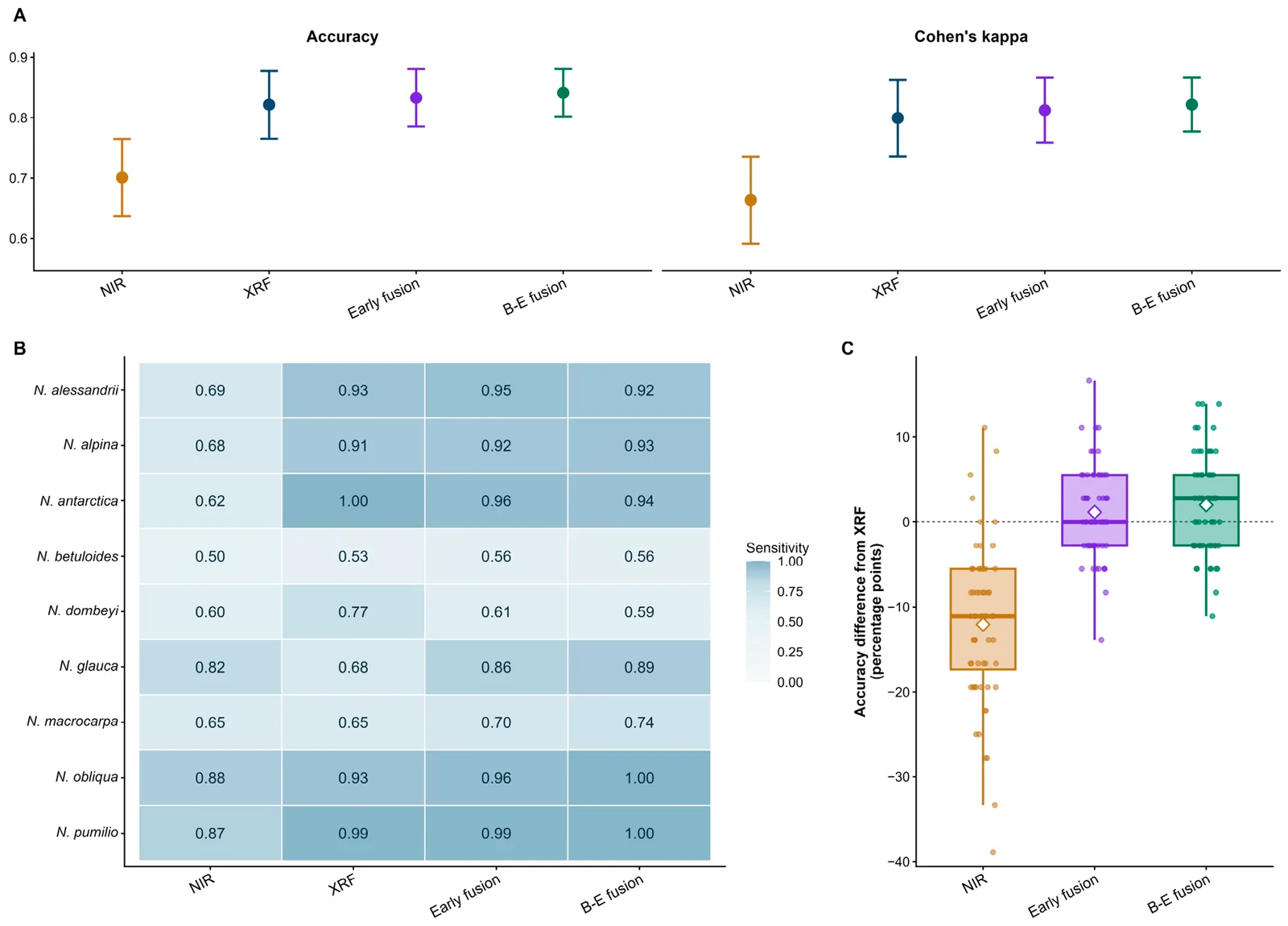
Performance of PLS-DA models under harmonized repeated internal cross-validation in the balanced nine-species dataset (n = 108 independent trees). (**A**) Mean accuracy and Cohen’s κ across stratified three-fold cross-validation repeated 20 times; error bars denote one SD across the 60 assessment partitions. (**B**) Mean class-wise sensitivity across the same partitions. (**C**) Paired partition-level differences in accuracy relative to XRF, expressed as percentage points; positive values indicate higher accuracy than XRF, and white diamonds denote means. The dashed horizontal line indicates zero difference from XRF. Partition-level contrasts are descriptive because assessment folds overlap across repeated cross-validation. B-E fusion, block-equalized fusion.

In the primary balanced analysis, the 14-component full-spectrum XRF model achieved an accuracy of 0.822 ± 0.056 (κ = 0.799 ± 0.063), compared with 0.701 ± 0.064 (κ = 0.664 ± 0.072) for the 13-component NIR model. Feature-scaled early fusion selected 21 components and achieved an accuracy of 0.833 ± 0.048 (κ = 0.812 ± 0.054), whereas block-equalized fusion selected 31 components and yielded 0.842 ± 0.040 (κ = 0.822 ± 0.045). Consensus accuracy followed the same ordering: 0.759 for NIR, 0.824 for XRF, 0.870 for early fusion and 0.889 for block-equalized fusion (Table 2; Figure 5A).

Partition-level contrasts showed modest and heterogeneous gains over XRF. Relative to XRF, mean accuracy differences were +1.16 percentage points for early fusion and +1.99 percentage points for block-equalized fusion. The corresponding empirical 2.5–97.5th percentile ranges included zero (−7.0 to 11.1 and −7.0 to 13.9 percentage points, respectively). Early fusion exceeded XRF in 45.0% of partitions and tied it in 23.3%, whereas block-equalized fusion exceeded XRF in 53.3% and tied it in 13.3%. Because assessment samples overlap across repeated folds, these paired distributions were interpreted descriptively rather than as formal inference (Figure 5C).

Taxon-level sensitivities further showed that fusion effects were not uniform (Figure 5B). Relative to XRF, block-equalized fusion increased mean sensitivity for *N. glauca* (0.68 to 0.89), *N. macrocarpa* (0.65 to 0.74) and *N. obliqua* (0.93 to 1.00), but decreased sensitivity for *N. dombeyi* (0.77 to 0.59). *N. betuloides* remained the least accurately resolved species across modalities (sensitivity = 0.50–0.56). The exploratory 10-taxon analysis, including the hybrid *N. × leoni*, retained the same performance ranking as the primary analysis, with accuracies of 0.661 ± 0.065 for NIR, 0.775 ± 0.045 for XRF, 0.800 ± 0.045 for early fusion and 0.805 ± 0.054 for block-equalized fusion; corresponding κ values were 0.622 ± 0.073, 0.749 ± 0.050, 0.777 ± 0.050 and 0.783 ± 0.060, respectively. Balanced accuracy showed the same ordering (0.631 ± 0.064, 0.739 ± 0.045, 0.773 ± 0.050 and 0.779 ± 0.058, respectively). Thus, inclusion of the underrepresented natural hybrid reduced overall performance but did not alter the relative ranking of the four modelling strategies.

### 2.4. Unsupervised O2PLS Reveals Substantial Shared NIR-XRF Taxon-Associated Structure

**Figure 6:**
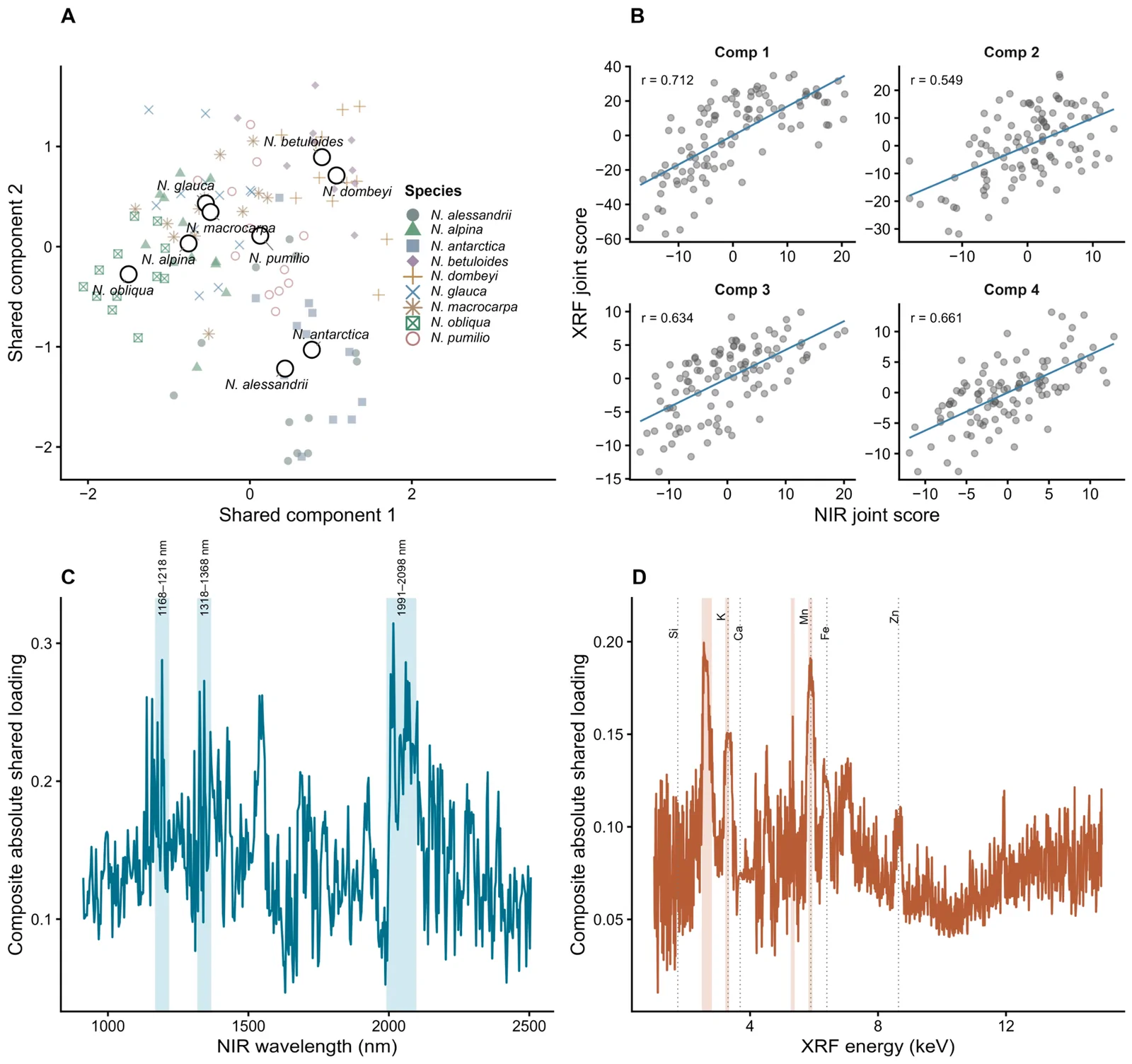
Shared and sensor-specific structure revealed by unsupervised O2PLS integration of NIR and XRF spectra in the balanced nine-species dataset (n = 108 independent trees). (**A**) Integrated shared-score space derived by sign-aligning and averaging standardized paired NIR and XRF joint scores; small symbols represent individual trees and open circles species centroids. Species labels were not used to estimate the O2PLS components. (**B**) Paired NIR and XRF scores for the four joint components; lines are ordinary least-squares fits and r values are Pearson correlations. (**C**) Composite absolute shared loading across the NIR domain, calculated as the sum of absolute joint loadings across the four joint components; shaded bands denote the strongest separated high-loading regions used for visualization. (**D**) Corresponding composite shared loading across the XRF domain; shaded bands denote the strongest separated high-loading regions, and dotted vertical lines mark theoretical Kα energies of Si, K, Ca, Mn, Fe and Zn as spectral references. Highlighted regions are descriptive and should not be interpreted as calibrated elemental concentrations or as direct one-to-one assignments between NIR bands and individual elements.

To determine whether multimodal complementarity reflected a common latent phenotype rather than only differences in supervised classification performance, the balanced nine-species NIR and XRF blocks were decomposed using unsupervised O2PLS. Cross-validation selected four joint components, four NIR-specific components and three XRF-specific components. Joint variation accounted for 45.8% of standardized NIR variation and 52.1% of standardized XRF variation, whereas sensor-specific components accounted for 22.1% and 22.8%, respectively; the remaining fractions were residual (Table 3). Paired NIR and XRF joint scores were consistently correlated across the four components (Pearson r = 0.712, 0.549, 0.634 and 0.661; Figure 6B), demonstrating substantial cross-modal covariation.

Although species identity was not used to estimate the O2PLS components, taxon-associated organization was strongest in the integrated shared space (PERMANOVA R^2^ = 0.605, pseudo-F = 18.96, *p* = 0.0001), compared with the NIR-specific (R^2^ = 0.212, pseudo-F = 3.33, *p* = 0.0001) and XRF-specific (R^2^ = 0.300, pseudo-F = 5.32, *p* = 0.0001) spaces (Table 3). Mean silhouette width was modestly positive in the shared space (0.056) but negative in the NIR-specific (−0.103) and XRF-specific (−0.099) spaces, indicating structured taxon displacement rather than complete separation into discrete clusters. Species centroids nevertheless occupied differentiated regions of the shared score map despite individual overlap (Figure 6A). Composite shared loadings were distributed across multiple NIR and XRF regions (Figure 6C,D), supporting a common cross-modal spectral phenotype together with additional modality-specific structure.

For interpretation, positive silhouette values indicate that observations are, on average, closer to their own taxon cluster than to the nearest alternative cluster, whereas negative values indicate greater proximity to another cluster.

## 3. Discussion

From a conservation perspective, the central finding is that a long-established living collection retains coherent taxon-associated phenotypic structure that can be recovered across complementary spectral domains. NIR and XRF measurements acquired from the same homogenized leaf material resolved different taxon-level arrangements and classification-error patterns, indicating that the two modalities captured complementary dimensions of leaf variation. Full-spectrum XRF provided the strongest stand-alone discrimination, supported by substantial interspecific differences across several element-associated signals. NIR was less accurate for taxonomic assignment but contributed nonredundant molecular-compositional and scattering-related information, while low-level fusion produced the highest overall point estimates. However, gains over XRF alone were modest, heterogeneous among validation partitions, and taxon dependent. The broader implication is therefore not that increasing predictor dimensionality necessarily improves classification, but that physically distinct spectral domains recover complementary components of phenotypic differentiation whose usefulness depends on the taxon and analytical objective.

The long-established common-garden living collection is central to this interpretation. The sampled trees had grown at the same site for approximately 18–25 years, reducing the immediate among-site environmental contrasts that complicate comparisons among geographically separated natural populations. However, the collection is not equivalent to a synchronized, randomized provenance trial: establishment years differed, provenance information was incomplete, and age, maternal, developmental, and local microenvironmental effects could not be separated formally. The shared site therefore provides evidence for persistent taxon-associated spectral differentiation among the sampled accessions, but it does not identify the causal basis of those differences or demonstrate genetic differentiation. Previous studies have shown that spectral phenotypes can recover molecular-marker-defined genetic groups in crop and forest-tree material, while common-garden experiments demonstrate that genetic identity, environment, and their interaction can remain jointly detectable in leaf reflectance [9,11,12]. In the present study, by contrast, no molecular data or replicated provenances were available to partition those sources of variation. Our inference is therefore restricted to persistent taxon-associated molecular-compositional, scattering-related, and element-associated spectral differences under broadly shared site conditions.

The modality-specific heatmaps and dendrograms showed that complementarity was already evident before supervised classification. This was particularly clear for *N. × leoni*, the natural hybrid between *N. obliqua* and *N. glauca* [27]: in the NIR dendrogram, it clustered with *N. glauca* (AU = 100, BP = 100), whereas in the XRF dendrogram, it clustered with *N. obliqua* (AU = 99, BP = 99). This pattern is best interpreted as contrasting phenetic affinities with the two parental species across modalities. These affinities are descriptive and could arise from physiological, structural, elemental-homeostatic, provenance-related, or genetic processes. Because the dendrograms are phenetic rather than phylogenetic and no molecular data were generated here, the pattern cannot be attributed to differential parental inheritance or admixture. More generally, spectral similarity may arise from shared ancestry, introgression, convergence in leaf traits, elemental economy, leaf habit, or combinations of these processes in a lineage with a documented history of hybridization and reticulate evolution [20].

Beyond these terminal affinities, the exploratory grouping analyses suggest that the two spectral domains also differ in how they reflect broader functional organization. Leaf habit was more clearly structured in XRF than in NIR, whereas subgeneric affiliation was evident in both modalities but again stronger in XRF. This contrast is biologically plausible because evergreen and deciduous strategies differ in leaf longevity, nutrient investment and nutrient-retention dynamics, traits that form part of the broader leaf economics spectrum [28]. Nutrient resorption during senescence is likewise closely linked to plant nutrient economy and differs among woody plant strategies, including evergreen and deciduous species [29,30]. Because XRF is directly sensitive to element-associated emission signals, differences in nutrient acquisition, retention, retranslocation and allocation could contribute to the stronger leaf-habit organization observed in this spectral domain. Recent ionomic work in Populus further demonstrates that leaf elemental profiles are biologically structured phenotypes with a substantial genetic component rather than simple reflections of soil chemistry alone [14].

By contrast, NIR integrates broad and partially overlapping information on biochemical composition and scattering-related properties, and trait-to-spectrum relationships vary among functional groups and biological contexts [6]. The weaker leaf-habit separation in NIR is therefore not unexpected and does not imply that leaf habit is absent from the NIR phenotype; rather, its signal may be distributed across multiple covarying chemical attributes instead of being expressed through a more discrete element-associated profile. Importantly, neither the leaf-habit nor subgeneric contrasts can be interpreted as independent effects because both attributes are structured by taxonomic identity in this collection. Their value is therefore primarily interpretative: they indicate that spectral differentiation among *Nothofagus* taxa is not arbitrary, but aligns partly with recognized functional strategies and evolutionary organization.

The XRF result is biologically plausible because the ionome operates as an integrated multielement system rather than as a set of independent concentrations. Mineral acquisition, transporter activity, root-microbe interactions, vascular allocation, leaf longevity and homeostatic regulation jointly shape elemental composition [13,14,31]. Under a shared site, species-associated differences in these processes can generate persistent multielement profiles. Fe, Zn, Ca and Mn displayed the largest taxon effects, while Mn, Fe, K and Si contributed most strongly to reduced-feature PLS-DA. The weaker and less stable P response is also instructive: statistical significance at the full taxon set did not persist in the balanced sensitivity analysis, underscoring the value of separating robust elemental differentiation from marginal findings.

The eight-signal model nevertheless performed substantially below the full XRF spectrum. This difference indicates that discrimination was not confined to the dominant integrated peaks. Whole-spectrum models can exploit peak shape, shoulders, secondary lines, continuum structure and coordinated weak signals that are lost when spectra are compressed into a small number of peak areas. Some of that additional information may reflect genuine multielement patterning, whereas some may arise from matrix and scattering effects associated with the standardized powders. The full spectrum should therefore be described as a powerful spectral fingerprint rather than as a direct multielement concentration profile. Peak areas were background corrected and analyzed under standardized preparation, but absorption, particle size, packing density, thickness and geometry can still affect XRF response [15,16,17,32]. Absolute concentration claims require matrix-matched calibration or comparison with ICP-based assays.

NIR represented a more continuous and distributed phenotype. Broad bands across approximately 896–2515 nm integrate many covarying chemical constituents and physical scattering responses, which can reduce separability among closely related taxa, even when biologically meaningful variation is present. The lower NIR accuracy should therefore not be interpreted as analytical failure. Reflectance spectra have captured genetic and phylogenetic structure in oaks and across seed plants, but the strength and location of informative signals vary among lineages, environments and experimental designs [6,8,33]. In the present data, NIR resolved several terminal associations with strong bootstrap support and contributed enough nonredundant information to alter fusion sensitivities, but it did not match the taxonomic discrimination achieved by XRF.

The O2PLS decomposition clarifies why the two modalities can be complementary despite their different predictive accuracies. Nearly half of the standardized variation in each block was captured by joint components (45.8% for NIR and 52.1% for XRF), and the paired joint scores showed moderate-to-strong cross-modal correlations. Because species labels were not used to derive the O2PLS latent spaces, the high taxon-associated variation in the shared representation (R^2^ = 0.605) provides independent descriptive evidence that much of the NIR-XRF covariation is biologically structured rather than solely a consequence of supervised classification. At the same time, the NIR-specific and XRF-specific spaces retained significant taxonomic organization (R^2^ = 0.212 and 0.300, respectively), indicating that complementarity comprises both common and modality-specific dimensions. The low silhouette values caution against interpreting the shared space as nine discrete clusters; instead, taxa are displaced along overlapping latent gradients. Likewise, shared NIR and XRF loading regions should be interpreted as covarying spectral domains, not as evidence that NIR directly detects individual elements.

The fusion analysis requires a deliberately restrained interpretation. Feature-scaled and block-equalized fusion increased balanced-analysis accuracy by only 1.16 and 1.99 percentage points relative to XRF, and empirical partition-level ranges included zero. Block equalization improved mean sensitivity for *N. glauca*, *N. macrocarpa* and *N. obliqua* but reduced it for *N. dombeyi*, while *N. betuloides* remained difficult across all modalities. Thus, the fused models changed the error landscape rather than uniformly improving every class. Block equalization is itself an analytical weighting choice: assigning equal expected total variance to the standardized NIR and XRF blocks changes the geometry of the fused predictor space and can redistribute classification errors among taxa. The reduced sensitivity of *N. dombeyi* illustrates this risk. The slightly higher point estimate and lower resampling SD observed here should therefore be treated as dataset-specific rather than as evidence that block equalization will improve performance generally. External validation is needed to determine whether the small internal advantage persists across independent trees, collections, years, instruments, and sites.

Although replication of 12 independent trees per species in the balanced analysis may appear modest for capturing the full breadth of intraspecific variation, the study was designed to characterize coherent taxon-level differentiation within the living collection rather than to estimate species-wide diversity. Importantly, the biological signal was supported by several convergent lines of evidence: modality-specific species-mean structure, strongly supported terminal clusters, repeated cross-validated discrimination, and substantial interspecific effects across multiple element-associated XRF signals. This convergence indicates that taxonomic differentiation was not an artefact of a single analytical procedure. Such an interpretation is consistent with emerging spectral-biology frameworks, which view spectra as integrative phenotypes capable of connecting taxonomic identity with biochemical, structural, and functional variation across biological scales [5]. It also accords with recent common-garden evidence showing that leaf reflectance retains detectable genetic, environmental, and genotype-by-environment components in forest trees [9]. Thus, despite moderate within-species replication, the present study provides a biologically coherent and analytically replicated characterization of taxonomic and elemental differentiation under shared site conditions, while avoiding claims about species-wide phenotypic diversity.

All predictive metrics in this study quantify internal discrimination within a single living collection and one sampling campaign; they are not estimates of transferability to other gardens, years, provenances, instruments, or wild populations. Living collections can provide exceptional taxonomic coverage and long-term research infrastructure, but their accessions do not necessarily capture the provenance structure or within-species diversity present in the wild [26]. In our study, *N. × leoni* was represented by six independent trees, whereas the three *N. nitida* spectra were analytical replicates derived from one biological tree; consequently, the balanced nine-species analysis provides the primary benchmark for predictive performance. The absence of replicated provenances and synchronized establishment further prevents formal separation of taxon, provenance, age, and persistent developmental effects. Sampling was also restricted to 8–9 January 2025, so the observed spectral patterns represent leaf status during one austral-summer phenological period and do not characterize seasonal variation. Finally, the XRF features represent standardized relative signals rather than calibrated elemental concentrations. Recent common-garden ionomic research in *Populus*, based on quantitative elemental platforms and hundreds of genotypes, has demonstrated a substantial genetic contribution to leaf elemental composition [14]; our findings are compatible with biologically structured elemental phenotypes but should be regarded as comparative micro-XRF fingerprinting rather than quantitative ionomics.

Taken together, these findings position paired NIR and micro-XRF spectral profiling as a framework for characterizing complementary taxon-associated variation in living collections. Within this collection, full-spectrum XRF provided the strongest stand-alone taxon discrimination, whereas NIR contributed complementary molecular-compositional and scattering-related information and produced modest additional gains when integrated with XRF. The value of multimodal acquisition therefore lies less in automatically replacing a strong single modality than in broadening the biological dimensions recovered from the same standardized material. Any operational use for taxonomic verification, germplasm auditing, or conservation decision-making will require provenance-resolved sampling and external validation across independent collections, seasons, and analytical settings.

## 4. Materials and Methods

### 4.1. Study System and Long-Established Common-Garden Living Collection

The study was conducted at Parque VerdeNativo, Puerto Octay, Los Lagos Region, southern Chile (40.988188° S, 72.913547° W; 108 m a.s.l.). The arboretum was initiated in 2000 as an ex situ conservation collection and contains the complete set of *Nothofagus* taxa native to Chile [34]. Seeds were progressively obtained from nurseries and private donations originating from different parts of Chile, germinated, and raised in a nursery on the same property before planting. The collection was therefore assembled opportunistically rather than through systematic sampling of predefined provenances.

*Nothofagus dombeyi* was planted in 2000 and the remaining taxa were progressively established through approximately 2007. The sampled trees had consequently grown at the site for about 18–25 years before leaf collection in January 2025. Because establishment was not synchronous and the collection was not designed as a randomized provenance trial, “common-garden living collection” denotes prolonged growth at one site rather than a balanced genetic experiment. The design reduces among-site environmental contrasts but does not eliminate inherited provenance effects, maternal effects, age differences or persistent developmental responses.

### 4.2. Study Taxa, Sampling Design and Sample Preparation

The collection comprised 11 taxa native to Chile and adjacent Argentina: *Nothofagus alessandrii*, *N. alpina*, *N. antarctica*, *N. betuloides*, *N. dombeyi*, *N. glauca*, *N. × leoni*, *N. macrocarpa*, *N. nitida*, *N. obliqua*, and *N. pumilio*. The aligned analytical matrix contained 117 sample units: 12 for each taxon except *N. × leoni* (n = 6) and *N. nitida* (n = 3). The three *N. nitida* spectra were technical measurements derived from leaf material collected from the same individual tree and were therefore not biologically independent. They were retained only for descriptive taxon-level analyses, including species-mean spectral summaries, heatmaps, and dendrograms. Predictive analyses were restricted to biologically independent trees and comprised two complementary datasets. The primary balanced analysis included nine non-hybrid species represented by 12 independent trees each (n = 108). A complementary exploratory analysis additionally included the naturally occurring hybrid *N. × leoni* (n = 6), yielding ten taxa and 114 biologically independent trees. Throughout the manuscript, ‘trees’ denotes biological replicates, whereas ‘spectra’ or ‘technical measurements’ denotes repeated analytical measurements of a biological sample.

Leaf material was collected on 8–9 January 2025. Approximately 2 kg of foliage was sampled per tree from multiple branches on the northeastern side of the mid-crown, at approximately 2–3 m above ground, in trees growing under closed-canopy conditions. Sampling targeted mature, fully expanded leaves. Taxonomic identity followed the curated botanical labels maintained in the living collection and was checked by the research team before sampling. Samples were placed in cooled containers immediately after harvest and transported under refrigeration to the University of Talca. Leaves were oven-dried at 50 °C for 48 h and subsequently ground manually using a medium-sized mortar to obtain a fine, homogenized powder. Powders were stored dry in sealed containers until analysis. The same homogenized powder from each sample unit was used for NIR and micro-XRF acquisition, preventing modality comparisons from being confounded by different leaf subsamples. Because all trees were sampled during a single two-day period in austral summer, the resulting spectral fingerprints represent leaf status at that sampling period rather than seasonal or phenological variation through the year.

### 4.3. NIR Spectral Acquisition and Preprocessing

NIR spectra were acquired in reflectance mode using a NIRQuest512 spectrometer (Ocean Insight, Orlando, FL, USA) coupled to an HL-2000-HP-FHSA tungsten-halogen light source and a QR600-7-VIS-125F bifurcated fibre-optic probe (both Ocean Insight, Orlando, FL, USA; 3.18-mm probe diameter), following the standardized laboratory acquisition protocol used for foliar spectroscopy [35,36]. The lamp was allowed to stabilize for 10 min before calibration and measurement. Spectra were acquired in OceanView using an integration time of 100 ms and 10 scans averaged per saved spectrum. A certified Spectralon reflectance standard was used to establish the white reference, whereas the dark reference was recorded with the lamp shutter closed and the probe covered to exclude external light. During acquisition, samples were positioned at the same optical geometry and height used for the reflectance reference. Three technical spectra were recorded from each homogenized powder sample and subsequently averaged to obtain one representative spectrum per analytical unit. The recorded range was 895.915–2514.925 nm and comprised 512 wavelength variables.

Raw reflectance percentages were converted to proportional reflectance (R = reflectance/100) and then to pseudoabsorbance as log10(1/R). Candidate preprocessing strategies included pseudoabsorbance alone, standard normal variate (SNV), first- and second-order Savitzky–Golay derivatives and derivative-plus-SNV combinations [37,38]. The finalized pipeline used a first Savitzky–Golay derivative with an 11-point window and second-order polynomial, followed by removal of five wavelengths from each edge and SNV correction, retaining 502 predictors. Predictor centring and unit-variance scaling were estimated within each cross-validation training fold and applied to the corresponding assessment fold. The comparison among candidate spectral transformations formed part of model development using this same collection and was not nested within an independent outer validation loop. The archived analysis records do not support reporting a separate prespecified optimization metric for that preprocessing comparison. Accordingly, the repeated-cross-validation performance reported here represents internal validation and may be optimistic with respect to the choice of spectral preprocessing; it should not be interpreted as independent validation of the preprocessing pipeline.

### 4.4. Micro-XRF Acquisition and Full-Spectrum Preprocessing

Elemental spectra were acquired using a Bruker M4 TORNADO Plus micro-XRF spectrometer (Bruker Nano GmbH, Berlin, Germany) at the University of Talca, following established applications of this platform for plant-material elemental profiling [39]. The instrument was equipped with a rhodium X-Ray source and an energy-dispersive silicon drift detector. Homogenized leaf powders were placed in identical sample holders, levelled to a reproducible depth, and measured under a constant instrumental configuration. The X-Ray source was operated at 50 kV and 600 μA, using a 160 μm spot size and an acquisition time of 20 ms per point under atmospheric pressure; measurements were performed using the multipoint acquisition tool in Bruker ESPRIT 1.2 software (Bruker Nano GmbH, Berlin, Germany). Three technical measurements were obtained from each independently prepared powder sample and aggregated at the sample level before multivariate analysis. Full spectra were exported as counts without conversion to elemental concentrations; accordingly, the analyses represent comparative spectral fingerprints and relative element-associated signals rather than calibrated tissue concentrations.

The original spectrum contained 3907 channels spanning approximately 0.0049–39.0649 keV. Full-spectrum analyses were restricted a priori to 1–15 keV (actual retained range, 1.0049–14.9949 keV), yielding 1400 channels that encompassed the principal detectable plant-element region while excluding the unstable lowest-energy channels and the high-energy region with little biological signal. Counts were transformed as log(count + 1). Predictor centring and unit-variance scaling were fitted independently within each training fold.

### 4.5. Element-Resolved XRF Features and Analytical Quality Control

Candidate element-associated signals were defined around tabulated Kα transition energies [40]. A statistics-sensitive nonlinear iterative peak-clipping (SNIP) baseline was estimated and subtracted from each spectrum before peak integration. Corrected counts were integrated within approximately ±0.10 keV of the theoretical peak centre and transformed as log(area + 1). Peak regions and neighbouring spectral structure were visually and analytically inspected to identify low signal and interference from adjacent lines. These features are relative XRF signals and cannot be interpreted as absolute tissue concentrations without matrix-matched standards or validation against an independent elemental assay.

Thirteen candidate signals were initially considered: Na, Mg, Al, Si, P, S, Cl, K, Ca, Mn, Fe, Cu and Zn. Na, Mg, Al and Cu were not sufficiently resolved and were excluded. Cl was also excluded because its K-shell emission overlaps with rhodium L-shell emission from the X-Ray tube, preventing reliable attribution. Eight signals were retained for comparative inference and reduced-feature classification: Si, P, S, K, Ca, Mn, Fe and Zn.

### 4.6. Unsupervised Spectral Structure and Exploratory Biological Grouping

Cluster robustness was assessed with 10,000 multiscale bootstrap resamples in pvclust, reporting approximately unbiased (AU) and conventional bootstrap probability (BP) values; AU ≥ 95% was interpreted as strong support [41]. Dendrograms were interpreted as modality-specific phenetic summaries, not as phylogenetic reconstructions. Species-level spectral profiles were obtained by averaging finalized preprocessed values at each wavelength or energy channel. For heatmaps and phenetic comparisons, each channel was standardized across taxon means to zero mean and unit variance. Euclidean dissimilarities were calculated separately for NIR and XRF, and hierarchical clustering used Ward.D2 agglomeration.

Exploratory PERMANOVA was used to quantify broad organization by leaf habit and subgeneric affiliation. Leaf-habit organization was evaluated at the taxon level using one channel-wise standardized species-mean profile per taxon. The three *N. nitida* analytical spectra were averaged before analysis so that this taxon contributed a single taxon-level profile. Euclidean dissimilarities were analyzed separately for NIR, XRF and feature-scaled fusion. Because the dataset comprised eight deciduous and three evergreen taxa, statistical significance was assessed by exact randomization over all 165 possible label allocations preserving group sizes. Homogeneity of multivariate dispersion was evaluated using PERMDISP under the same exact allocation framework. Subgeneric affiliation followed Heenan and Smissen [42] and was evaluated from species-mean profiles after excluding the singleton Fuscospora taxon *N. alessandrii*, leaving the Lophozonia and *Nothofagus* groups. For this contrast, analyses were conducted separately for NIR, XRF and feature-scaled fusion using 9999 permutations, with multivariate dispersion assessed by PERMDISP [43]. Because both leaf habit and subgenus were invariant within taxa, these analyses were interpreted as broad group-level organization rather than as effects separable from taxonomic identity.

### 4.7. Element-Level Inference and Interpretable Classification

For each of the eight retained signals, interspecific differences were evaluated with Welch one-way analysis of variance, which does not assume equal within-taxon variances. *N. nitida* was excluded from these inferential tests because its three spectra represented analytical replicates of one biological sample. *p* values were adjusted across the eight tests using the Benjamini–Hochberg false-discovery-rate procedure. Effect size was expressed as η^2^, the proportion of feature variance attributable to taxon. Robustness was evaluated using Kruskal–Wallis tests and a balanced nine-species Welch sensitivity analysis.

The eight retained signals were also entered into a PLS-DA model under the same repeated-validation framework as the full spectra. This reduced model tested how much taxonomic information could be retained in an interpretable element-associated representation. Feature contributions were summarized by the variable-importance measure from the fitted PLS model and interpreted jointly with effect sizes and taxon-level distributions.

### 4.8. PLS-DA Classification and Repeated Internal Validation

PLS-DA was used as the common classifier because it accommodates strongly collinear, high-dimensional predictors within a single modelling framework [44]. Models were fitted with the pls engine through caret [45,46]. Stratified three-fold cross-validation was repeated 20 times, producing 60 assessment partitions. Three folds were chosen because the primary design provided 12 independent trees per species, yielding four trees per taxon in each assessment fold while retaining eight for training. Five- or ten-fold schemes would place only about 2–3 or 1–2 trees per taxon, respectively, in individual assessment folds, making class-wise sensitivity more unstable. Repetition characterizes variability across alternative partitions but does not increase the biological sample size or constitute independent validation. The number of latent components was selected from a 1–40 grid by maximum repeated-cross-validation accuracy within resampling, and centring and unit-variance scaling required by model fitting were estimated from training data only. Identical saved partitions were used for aligned NIR, XRF and fusion comparisons.

The primary predictive analysis comprised nine non-hybrid species represented by 12 independent trees each (n = 108), providing a fully balanced benchmark. A complementary exploratory analysis additionally included the naturally occurring hybrid *N. × leoni* (n = 6), yielding ten taxa and 114 biologically independent trees. *N. nitida* was excluded from all predictive analyses because its available spectra represented analytical replicates derived from a single biological sample. The same modelling pipeline, component-tuning procedure and repeated internal cross-validation framework were applied independently to both analysis sets. Performance was summarized by mean accuracy, Cohen’s κ, balanced accuracy and class-wise sensitivity across the 60 assessment partitions. Balanced accuracy was particularly informative for the exploratory analysis because *N. × leoni* had lower representation than the remaining taxa.

### 4.9. Low-Level Multimodal Fusion

Low-level early fusion was performed after matching normalized sample identifiers rather than relying on row order. The finalized NIR matrix (502 predictors) and XRF matrix (1400 predictors) were concatenated to create 1902 predictors. In feature-scaled early fusion, each predictor was centred and scaled within the training fold. A complementary block-equalized fusion analysis additionally weighted the standardized NIR and XRF blocks by 1/√502 and 1/√1400, respectively, before concatenation. The block-equalized model used the algebraically equivalent non-iterative kernel PLS algorithm for numerical stability. Both fusion models used the same saved partitions, component grid and performance summaries as the single-modality analyses.

### 4.10. Shared and Sensor-Specific NIR-XRF Decomposition

To separate cross-modal covariation from systematic variation unique to each modality, the primary balanced dataset (nine species, n = 108 independent trees) was analyzed using two-way orthogonal partial least squares (O2PLS) [47] implemented in OmicsPLS [48]. Finalized NIR (502 predictors) and XRF (1400 predictors) blocks were centred and scaled to unit variance before fitting. O2PLS was used as an unsupervised two-block integration model, so species identity was not used to estimate the latent components. The numbers of joint and block-specific components were selected in two stages. First, five-fold cross-validation using the adjusted-R^2^ criterion evaluated 1–4 joint components and 0–3 modality-specific components per block. The best solution was then refined by standard five-fold prediction-error cross-validation over a local grid surrounding that optimum, and the combination minimizing cross-validated mean squared error was retained. The final model comprised four joint components, four NIR-specific components, and three XRF-specific components.

For each block, O2PLS variation was partitioned into joint NIR-XRF, sensor-specific orthogonal and residual fractions. Pearson and Spearman correlations were calculated between paired NIR and XRF joint scores. For visualization of the common latent phenotype, paired joint scores were sign-aligned, standardized within component and averaged to obtain integrated shared scores. Euclidean distances in the shared, NIR-specific and XRF-specific score spaces were evaluated for taxon-associated organization using PERMANOVA with 9999 permutations; mean silhouette widths and between-species centroid distances were treated descriptively. Composite shared loading profiles were calculated as the sum of absolute joint loadings across the retained joint components. The separated high-loading intervals highlighted in Figure 6 were used only for visualization and were not treated as inferentially selected features.

### 4.11. Statistical Software and Reproducibility

Analyses were conducted in R version 4.5.3 [49]. Spectral preprocessing used prospectr version 0.2.10; PLS-DA and repeated stratified cross-validation used caret version 7.0.1 and pls version 2.9.0; PERMANOVA and PERMDISP used vegan version 2.7.5; hierarchical bootstrap analyses used pvclust version 2.2.0; two-block O2PLS integration used OmicsPLS version 2.1.0; silhouette summaries used cluster version 2.1.8.2; and figures were produced principally with ggplot2 version 4.0.3. All tests were two-sided with α = 0.05. Random seeds, normalized identifiers, preprocessing parameters, and cross-validation partitions were fixed before final model fitting.

## 5. Conclusions

Paired NIR and micro-XRF spectral profiling recovered complementary dimensions of taxon-associated variation in a long-established ex situ collection of South American *Nothofagus*. Full-spectrum XRF provided the strongest stand-alone discrimination within the collection, while NIR contributed nonredundant information and multimodal integration produced only modest, taxon-dependent gains in predictive accuracy. O2PLS further showed that a substantial fraction of variation was shared across modalities while modality-specific spaces retained additional taxon-associated structure. These findings support spectral phenotyping as an instrumental framework for comparative characterization of living collections, but they do not establish genetic differentiation or general taxonomic verification beyond the sampled accessions. Independent collections, provenance-aware sampling, repeated seasonal measurements, and external validation are required before the approach can be used operationally for germplasm auditing or conservation management.

## Figures and Tables

**Table 1:** Interspecific differences in statistics-sensitive nonlinear iterative peak-clipping (SNIP) background-corrected relative micro-XRF element-associated signals across ten *Nothofagus* taxa (*N. alessandrii*, *N. alpina*, *N. antarctica*, *N. betuloides*, *N. dombeyi*, *N. glauca*, *N. × leoni*, *N. macrocarpa*, *N. obliqua* and *N. pumilio*) *.

Element	F (df_1_, df_2_)	*p* Value	BH-Adjusted *p* Value	η^2^
Silicon	11.15 (9, 39.4)	2.13 × 10^−8^	2.84 × 10^−8^	0.487
Phosphorus	2.19 (9, 39.2)	0.0439	0.0439	0.198
Sulfur	6.10 (9, 39.7)	2.40 × 10^−5^	2.74 × 10^−5^	0.384
Potassium	12.98 (9, 39.5)	2.57 × 10^−9^	5.15 × 10^−9^	0.466
Calcium	19.55 (9, 39.3)	6.72 × 10^−12^	1.79 × 10^−11^	0.575
Manganese	11.75 (9, 40.7)	7.81 × 10^−9^	1.25 × 10^−8^	0.533
Iron	26.69 (9, 39.6)	3.94 × 10^−14^	2.66 × 10^−13^	0.720
Zinc	24.92 (9, 41.0)	6.66 × 10^−14^	2.66 × 10^−13^	0.691

Note. F, Welch test statistic; df_1_ and df_2_, numerator and denominator degrees of freedom; BH, Benjamini–Hochberg; η^2^, proportion of variance attributable to the taxon. Signals were transformed as log(area + 1) and represent relative features, not calibrated concentrations. * *Nothofagus nitida* was not included in inferential analyses because only one biological sample was available.

**Table 2:** Performance of PLS-DA models under harmonized repeated internal cross-validation in the primary balanced nine-species dataset and the exploratory 10-taxon dataset, including the natural hybrid *N. × leoni*.

Analysis Set	Modality	LVs	CV Accuracy	CV Cohen’s κ	CV Balanced Accuracy	Consensus Accuracy (κ)
Primary balanced: 9 species (n = 108)	NIR	13	0.701 ± 0.064	0.664 ± 0.072	0.701 ± 0.064	0.759 (0.729)
Primary balanced: 9 species (n = 108)	XRF	14	0.822 ± 0.056	0.799 ± 0.063	0.822 ± 0.056	0.824 (0.802)
Primary balanced: 9 species (n = 108)	Early fusion	21	0.833 ± 0.048	0.812 ± 0.054	0.833 ± 0.048	0.870 (0.854)
Primary balanced: 9 species (n = 108)	Block-equalized fusion	31	0.842 ± 0.040	0.822 ± 0.045	0.842 ± 0.040	0.889 (0.875)
Exploratory including hybrid: 10 taxa (n = 114)	NIR	13	0.661 ± 0.065	0.622 ± 0.073	0.631 ± 0.064	0.702 (0.667)
Exploratory including hybrid: 10 taxa (n = 114)	XRF	16	0.775 ± 0.045	0.749 ± 0.050	0.739 ± 0.045	0.798 (0.775)
Exploratory including hybrid: 10 taxa (n = 114)	Early fusion	30	0.800 ± 0.045	0.777 ± 0.050	0.773 ± 0.050	0.833 (0.814)
Exploratory including hybrid: 10 taxa (n = 114)	Block-equalized fusion	27	0.805 ± 0.054	0.783 ± 0.060	0.779 ± 0.058	0.851 (0.834)

Note: LVs, latent variables; CV, cross-validation; κ, Cohen’s kappa. CV metrics are means ± SD across 60 assessment partitions from stratified three-fold cross-validation repeated 20 times, using identical saved partitions across modalities within each analysis set. The primary analysis comprised nine non-hybrid species represented by 12 independent trees each (n = 108). The exploratory analysis additionally included *N. × leoni* (n = 6), yielding 10 taxa and 114 biologically independent trees. Components were selected from a 1–40 grid within repeated internal cross-validation. Balanced accuracy is reported because the exploratory analysis is unbalanced with respect to *N. × leoni*. Consensus values are accuracy (Cohen’s κ) from modal repeated out-of-fold predictions, with ties resolved using mean taxon probability.

**Table 3:** Orthogonal two-block partial least squares (O2PLS) decomposition of shared and sensor-specific variation between NIR and XRF spectral blocks and taxon-associated organization of the corresponding latent spaces in the balanced nine-species dataset (n = 108 independent trees). Taxonomic organization was assessed using PERMANOVA and mean silhouette width. Positive silhouette values indicate that observations are, on average, closer to their own taxon cluster than to the nearest alternative cluster; negative values indicate greater proximity to another cluster.

O2PLS Component	NIR Variance (%)	XRF Variance (%)	PERMANOVA R^2^	*p*-Value	Mean Silhouette Width
Shared predictive structure	45.8	52.1	0.605	<0.001	0.056
NIR-specific orthogonal structure	22.1	-	0.212	<0.001	−0.103
XRF-specific orthogonal structure	-	22.8	0.300	<0.001	−0.099
Residual unexplained variation	32.1	25.0	-	-	-

## Data Availability

The data presented in this study are openly available in the Science Data Bank (ScienceDB) at https://doi.org/10.57760/sciencedb.010dg.
